# Research priorities for idiopathic epilepsy in dogs: Viewpoints of owners, general practice veterinarians, and neurology specialists

**DOI:** 10.1111/jvim.16144

**Published:** 2021-05-07

**Authors:** Gareth Michael Couper Jones, Holger Andreas Volk, Rowena Mary Anne Packer

**Affiliations:** ^1^ Department of Clinical Science and Services Royal Veterinary College Hertfordshire UK; ^2^ Department of Small Animal Medicine and Surgery University of Veterinary Medicine Hannover Hannover Germany

**Keywords:** antiepileptic medication, dog, quality of life, veterinary neurology

## Abstract

**Background:**

Epilepsy is the most common chronic neurological disease in dogs that adversely affects the quality of life (QoL) of affected dogs and their owners. Research on epilepsy in dogs is expanding internationally, but where best to focus limited research time, funds, and expertise to achieve better outcomes for affected dogs and their owners has not been studied.

**Objective:**

To explore idiopathic epilepsy (IE) research priorities of owners of dogs with IE, general practice veterinarians, and veterinary neurologists.

**Methods:**

An international online survey was conducted in 2016 and repeated in 2020. Participants rated the absolute importance and relative rank of 18 areas of IE research, which were compared between groups and time points.

**Results:**

Valid responses were received from 414 respondents in 2016 and 414 respondents in 2020. The development of new anti‐seizure drugs (ASD) and improving the existing ASD management were considered the most important research priorities. Areas of research with increasing priority between 2016 and 2020 included non‐ASD management, with the greatest potential seen in behavioral and dietary‐based interventions. Disagreements in priorities were identified between groups; owners prioritized issues that impacted their and their dog's QoL, for example, adverse effects and comorbidities, whereas general practitioner vets and neurologists prioritized clinical issues and longer‐term strategies to manage or prevent IE, respectively.

**Conclusions and Clinical Importance:**

Ensuring that voices of owners are heard in the planning of future research should be a broader goal of veterinary medicine, to target research efforts toward areas most likely to improve the QoL of the dog‐owner dyad.

**Abbreviations**
ASD: anti‐seizure drug
CBD: cannabidiol
CRISPR: clustered regularly interspaced short palindromic repeats
DBS: deep brain stimulation
ECVN: European College of Veterinary Neurologists
GP: general practitioner
IE: idiopathic epilepsy
IQR: interquartile range
IVETF: International Veterinary Epilepsy Task Force
MCT: medium‐chain triglyceride
QoL: quality of life
TMS: transcranial magnetic stimulation
VNS: vagus nerve stimulation

## 1 INTRODUCTION

Idiopathic epilepsy (IE) is the most common chronic neurological disorder in dogs, defined as epilepsy with an unknown, genetic, or suspected genetic origin,^1^ and affects 0.6% to 0.7% of dogs.^2^, ^3^ Although IE is by definition a seizure disorder in both dogs^1^ and people,^4^ IE is characterized by more than recurrent epileptic seizures alone. An influx of studies on IE in dogs over the past 30 years highlights the complexity of IE as a general brain disease in dogs. Dogs with IE are not only affected by the ictal (seizure) episode itself, but can also experience a pre‐ictal “prodromal” phase minutes to hours before the seizure,^5^ and a post‐ictal phase, lasting minutes to days after the seizure.^6^ In addition to these peri‐ictal changes, recent evidence suggests that many dogs with IE exhibit inter‐ictal behavioral comorbidities including anxiety and fear,^7^, ^8^, ^9^ and ADHD‐like behavior.^10^, ^11^ Furthermore, there are cognitive impairments in dogs with IE, particularly related to learning and memory.^12^, ^13^, ^14^, ^15^ Combined, these effects reduce both quality of life (QoL) in affected dogs^16^, ^17^, ^18^, ^19^ and their caregivers,^20^, ^21^ and lead to a shortened life expectancy in affected dogs.^22^, ^23^

Given these potentially severe impacts, a large proportion of recent research efforts regarding IE in dogs have focused on developing therapies to reduce seizure frequency and severity,^24^ with an aim of dogs reaching remission (seizure freedom), or an ≥50% reduction in seizure frequency.^25^ Unfortunately, despite a range of potentially efficacious therapies, more than two thirds of dogs with IE continue to have epileptic seizures long‐term^22^, ^26^, ^27^, ^28^ and around 20% to 30% remain poorly controlled (<50% reduction of seizure frequency) despite treatment with phenobarbital, potassium bromide, or both treatments.^29^, ^30^, ^31^ Seizure freedom is rare, with just 14% of anti‐seizure drug (ASD)‐treated dogs entering remission.^32^ Alongside the challenges of drug‐resistance, many dogs treated with ASDs experience adverse effects including ataxia, lethargy, and polyphagia, which have the potential to impair QoL.^16^, ^33^ In response to these limitations, nondrug management options have been developed in recent years, including diet, surgery, and neurostimulation, with many of these methods adapted from human medical treatment of IE.^34^

Given this backdrop of broad challenges associated with the impact and management of IE in dogs, combined with limited time, expertise, and funds to conduct research to improve the welfare of affected dogs and their owners, prioritizing future research activities could focus future efforts toward the most needed areas. In human epilepsy research, organizations such as the International League Against Epilepsy have conducted prioritization activities to highlight the most important and urgent research needs,^35^, ^36^ along with similar activities from regional research networks that have included patient representatives as well as epilepsy researchers and clinicians.^37^, ^38^ The aims of the study were 2‐fold: first, to compare the future research priorities for IE in dogs between owners of dogs with IE (“owners”), specialist veterinary neurologists (neurologists) and general practice veterinarians (GP vets), and whether these priorities change over time; and second, to investigate perceptions of the impacts of emerging nondrug therapies upon the management of IE in dogs in 2020.

## 2 MATERIALS AND METHODS

### 2.1 Survey design

An online survey was designed in SurveyMonkey (Palo Alto, California) and was originally deployed from May to September 2016, with the second iteration deployed between May and September 2020.

### 2.2 Recruitment

Respondents were recruited from 3 defined groups:

1. Owners of dogs diagnosed with IE, either alive or deceased, who had been diagnosed as per the International Veterinary Epilepsy Task Force (IVETF) tier I criteria.^39^
2. Veterinarians who identified as general/primary care/first opinion practitioners (GPs).
3. Veterinarians with specialist qualifications in veterinary neurology (American College of Veterinary Internal Medicine or European College of Veterinary Neurologists [ECVN] Diplomates).

Respondents were recruited via several routes including social media (Facebook, Twitter), with owners specifically targeted via online support forums, vets via veterinarian‐specific websites, for example, Vet‐Surgeon.org, and neurology specialists via LISTSERVS for the 2 specialist colleges.

### 2.3 Rating and ranking of research areas

The survey compared the rating (ie, absolute rating of importance) and ranking (ie, relative importance) of 18 areas of IE research which were identified by the study team from peer‐reviewed IE studies before 2016 and research areas identified as priorities in human epilepsy. The areas included are listed in Table 1. Respondents were asked to assign an absolute importance rating to each area on a scale of 1 to 5, from 1 (no importance) to 5 (top priority) followed by a relative ranking from 1 to 18, from 1 (top priority) to 18 (least priority). The list of research areas was presented in a randomized order for each respondent for both rating and ranking, with ranking of areas presented as “drag and drop” boxes where tied ranks were not allowed, and ranking could be easily visualized.

**TABLE 1:** Research areas assigned absolute importance ratings and relative ranks by participants (general practice [GP] vets, neurologists, and owners of dogs with epilepsy), their variable label throughout the study, and their general area of research

Priority question	Variable label	Priority area
Improving existing drug management of epilepsy	Existing AEDs	Treatment
The adverse effects of seizure medication and why they occur	Adverse effects of AEDs	
Development of new antiepileptic medication	New AEDs	
Nondrug management of epilepsy such as diet, etc.	Non‐AED management	
How different types of seizures are classified, to help personalize therapies/management	Seizure classification	
Ways to detect seizures through development of wearable technology	Seizure detection	Diagnosis and detection
Ways epilepsy can be better and more quickly diagnosed	Diagnosing epilepsy	
Identifying the genetic causes of idiopathic epilepsy	Genetic etiology	
What are the nongenetic causes for idiopathic epilepsy	Nongenetic etiology	
The impact of epilepsy on dogs' anxiety	Anxiety	Behavioral comorbidities
The impact of epilepsy on dogs' hyperactivity	Hyperactivity	
The impact of epilepsy on dogs' physical capabilities	Physical capabilities	
The impact of epilepsy on dogs' attention/concentration levels	Attention	
How epilepsy affects social interaction of affected dogs	Social interactions	
The effect of epilepsy on other diseases (both pre‐existing and new conditions)	Comorbidities	Physical comorbidities
Ways to improve the education of vets regarding idiopathic epilepsy	Vet education	Education
The impact of epilepsy on dogs' lifespans	Lifespan	Outcomes
What epilepsy means in terms of prognosis	Prognosis	

### 2.4 Respondent demographics

All respondents were asked to report characteristics including group membership (owner of a dog diagnosed with IE, GP vet, or neurologist), country of residence, sex, and age.

### 2.5 Follow‐up survey

Based on the increase in publications and studies regarding nondrug therapies for IE between 2016 and 2020, an additional question was added in 2020 asking all respondents to report the potential for 10 nondrug therapies to positively impact upon the management of IE in dogs, on a scale of 0 (no impact), 1 (little impact), 2 (some impact), 3 (great impact), 4 (major impact), with an option of “I don't know what this is.” The therapies explored were: cannabidiol (CBD) oil supplementation, medium‐chain triglyceride (MCT) oil supplementation, raw food diet, hypoallergenic diet, vagus nerve stimulation (VNS), deep brain stimulation (DBS), gene editing, behavioral management, for example, lifestyle changes, seizure trigger avoidance, transcranial magnetic stimulation (TMS), and epilepsy surgery, for example, removal of seizure‐causing areas in the brain.

### 2.6 Statistical analysis

All statistics were conducted using software SPSS Statistics^26^ (IBM Corporation, New York). Categorical variables (eg, 1‐5 importance rating) are expressed as percentages and compared between years and respondent groups using the Chi‐squared test. Ordinal data (eg, ranking from 1 to 18) are expressed as median (interquartile range [IQR]) and compared between years and respondent groups using the Kruskall‐Wallis test, with pairwise Mann‐Whitney *U* tests where differences between groups were detected (with resultant *P* values adjusted by the Bonferroni correction for multiple tests). Results where *P* < .05 were considered statistically significant.

## 3 RESULTS

### 3.1 Respondent demographics

Valid responses were received from 414 respondents in 2016 (n = 302 owners, n = 84 GP vets, n = 28 neurologists) and 414 respondents in 2020 (n = 273 owners, n = 68 GP vets, n = 73 neurologists). Because of the sampling strategy, response rates could not be calculated. Specific demographics of each group are reported in Table 2. The 2016 and 2020 samples did not significantly differ with the exception of GP vet demographics, who were younger and less likely to be UK‐based in the 2020 sample. In 2016, 37.1% of owners had attended both their regular vet and a neurology specialist to diagnose and manage their dog's IE, and in 2020, this rose to 47.0% of owners.

**TABLE 2:** Demographic characteristics of study sample of respondents (general practice [GP] vets, neurologists, owners of dogs with epilepsy) compared across the 2016 and 2020 samples

Group	Variable	Subcategory	Overall	2016	2020	Year comparison	
			n (%)	n (%)	n (%)	*X* ^2^	*P*
GP Vet	Sex	Female	108 (72.0)	65 (77.4)	43 (65.2)	2.80	.25
		Male	40 (26.7)	18 (21.4)	22 (33.3)		
	Age	18‐30	67 (44.7)	28 (33.3)	39 (59.1)	15.20	.01
		31‐45	51 (34.0)	30 (35.7)	21 (31.8)		
		46‐60	27 (18.0)	22 (26.2)	5 (18.0)		
	Country	UK	106 (70.7)	71 (84.5)	35 (53.0)	17.70	<.001
		Non‐UK	44 (29.3)	13 (15.5)	31 (47.0)		
Neurologist	Sex	Female	57 (56.8)	17 (60.7)	40 (54.8)	0.30	.59
		Male	44 (43.6)	11 (39.3)	44 (45.2)		
	Age	18‐30	11 (10.9)	1 (3.6)	10 (13.7)	4.90	.18
		31‐45	59 (58.4)	16 (57.1)	43 (58.9)		
		46‐60	28 (27.7)	11 (39.3)	17 (23.2)		
	Country	UK	23 (22.8)	10 (35.7)	13 (17.8)	3.70	.06
		Non‐UK	78 (77.2)	18 (64.3)	60 (82.2)		
Owner	Sex	Female	530 (92.2)	279 (92.1)	252 (92.3)	0.80	.66
		Male	41 (7.1)	21 (7.0)	20 (7.3)		
	Age	18‐30	83 (14.4)	41 (13.6)	42 (14.4)	2.90	.72
		31‐45	190 (33.0)	95 (31.5)	95 (34.8)		
		46‐60	215 (37.4)	122 (40.4)	93 (34.1)		
		61‐75	82 (14.3)	42 (13.9)	40 (14.7)		
	Country	UK	273 (47.5)	164 (54.3)	138 (50.5)	0.80	.37
		Non‐UK	302 (52.5)	138 (45.7)	135 (49.5)		

Abbreviation: *X* ^2^, Chi‐squared test statistic.

### 3.2 Dog demographics and clinical characteristics

The most common breeds represented in both 2016 and 2020 samples were crossbreeds, Border Collies, Labrador Retrievers, and German Shepherd Dogs, with male neutered dogs the most common sex in both samples. There was no difference in any signalment feature between 2016 and 2020 samples (Table 3).

**TABLE 3:** Demographic characteristics of dogs diagnosed with epilepsy compared across the 2016 and 2020 samples. No signalment variables differed between time points

Variable	Subcategory	Overall	2016	2020	Year comparison	
		n (%)	n (%)	n (%)	*X* ^2^	*P*
Breeds (most common)	Crossbreed	131 (22.8)	74 (24.5)	57 (20.9)	107.23	.29
	Border Collie	65 (11.3)	31 (10.3)	34 (12.5)		
	Labrador Retriever	38 (6.6)	16 (5.3)	22 (8.1)		
	German Shepherd Dog	23 (4.0)	11 (3.6)	12 (4.4)		
Pedigree	Yes	260 (45.2)	134 (44.4)	126 (46.2)	0.75	.69
	No	292 (50.8)	154 (51.0)	138 (50.5)		
	I don't know	23 (4.0)	14 (4.6)	9 (3.3)		
Sex	Female entire	28 (4.9)	13 (4.3)	15 (5.5)	3.70	.30
	Female neutered	166 (28.9)	88 (29.1)	78 (28.6)		
	Male entire	92 (16.0)	41 (13.6)	51 (18.7)		
	Male neutered	289 (34.9)	160 (53.0)	129 (47.3)		
Variable	Subcategory	Mean ± SD	Mean ± SD	Mean ± SD	*t*	*P*
Age (months)	N/A	68.24 ± 1.45	68.18 ± 1.99	68.29 ± 2.13	0.04	.97

### 3.3 Future research priorities

#### 3.3.1 Rating of perceived importance

When owners, GP vets, and neurologists rated priorities using the scale of 1 (no importance) to 5 (top priority), research areas with the highest perceived importance were improving existing drug management of IE, development of new antiepileptic medication, and ways to improve the education of vets regarding IE (Table 4).

**TABLE 4:** Owner, general practice (GP) vet, and neurologist perceived importance of future research priorities for idiopathic epilepsy (IE) in dogs. Research areas were rated on a scale of 1 (no importance) to 5 (great importance) and the % of respondents in each rating category are stated for 2016 and 2020

Research area	2016						2020						Year comparison	
	N	1	2	3	4	5	N	1	2	3	4	5	*X* ^2^	*P*
Existing AEDs	407	0.2	2.2	9.1	31.9	56.5	412	0.5	2.7	7.5	35.0	54.4	1.83	.77
New AEDs	400	0.5	2.3	12.0	32.5	52.8	408	0.7	2.9	14.2	30.4	51.7	1.64	.80
Vet education	402	0.2	2.7	14.9	37.8	44.3	412	0.7	2.9	11.9	38.8	45.6	2.51	.64
Genetic etiology	403	0.5	6.5	15.9	33.7	43.4	411	1.0	8.8	21.7	31.1	37.5	7.87	.10
Adverse effects of AEDs	404	0.7	3.0	16.8	39.1	40.3	413	1.0	2.4	18.9	49.2	28.6	13.73	.01
Non‐AED management	406	1.5	6.4	18.5	41.9	31.8	411	1.2	5.8	19.5	39.9	33.6	0.71	.95
Seizure detection	408	0.7	10.0	28.7	34.6	26.0	410	1.5	7.8	22.2	40.5	28.0	7.76	.10
Diagnosing epilepsy	405	0.2	6.7	19.0	40.7	33.3	413	0.7	9.2	24.9	36.3	28.8	8.26	.08
Nongenetic etiology	403	0.5	5.2	20.3	39.7	34.2	412	1.0	7.8	23.3	42.5	25.5	9.11	.06
Seizure classification	405	0.7	7.7	28.6	42.7	20.2	412	0.7	8.5	23.3	45.6	21.8	3.07	.55
Lifespan	406	1.5	10.1	25.4	39.7	23.4	409	1.0	8.3	28.9	36.9	24.9	2.63	.62
Prognosis	410	1.2	6.8	38.5	42.2	21.2	413	1.0	7.3	27.4	43.6	20.8	0.38	.10
Comorbidities	399	0.5	11.0	31.1	41.1	16.3	406	1.0	11.8	34.7	39.4	13.1	3.14	.54
Anxiety	409	1.7	14.2	26.9	36.7	20.5	409	1.0	14.4	29.3	38.6	16.6	4.15	.53
Hyperactivity	401	4.7	20.2	35.2	26.4	13.5	401	3.5	27.9	37.4	24.2	7.0	14.66	.01
Physical capabilities	410	2.7	14.9	33.7	32.2	16.6	412	1.2	18.0	29.4	39.3	12.1	10.42	.03
Attention	413	3.9	20.3	35.4	27.4	13.1	408	3.4	25.5	40.0	21.1	10.0	8.61	.07
Social interactions	405	3.5	22.5	34.3	28.9	10.9	409	2.7	26.7	37.7	22.7	10.3	5.52	.24

Abbreviation: *X* ^2^, Chi‐squared test statistic.

Three research areas differed in importance between 2016 and 2020: the adverse effects of seizure medication and why they occur, which was rated significantly less important in 2020 than 2016, and the impact of IE on dogs' hyperactivity and physical capabilities, which was rated significantly more important in 2020 than 2016 (Table 4).

Differences in importance ratings between respondent groups were identified for 16 of the 18 research areas, with only how different types of epileptic seizures are classified and what IE means in terms of prognosis not significantly differing between groups (Table 5).

**TABLE 5:** Differences in the perceived importance of future research priorities for IE in dogs among neurologists (n = 101), general practice (GP) vets (n = 152), and owners (n = 575). Research areas were rated on a scale of 1 (no importance) to 5 (great importance). Data from 2016 and 2020 are pooled

Research area	Owners (%)					Neurologists (%)					GP vets (%)					Statistical test	
	1	2	3	4	5	1	2	3	4	5	1	2	3	4	5	*X* ^2^	*P*
Existing AEDs	0.2	2.1	6.3	32.3	59.1	0.0	3.0	13.9	34.7	48.5	1.3	3.3	11.9	37.1	46.4	19.64	.01
New AEDs	0.5	2.2	10.2	29.8	57.3	0.0	2.0	9.0	34.0	55.0	1.3	4.6	26.5	35.8	31.8	46.79	<.001
Vet education	0.2	1.4	9.1	35.7	53.6	1.0	6.0	23.0	45.0	25.0	1.3	6.0	23.2	43.7	25.8	76.03	<.001
Adverse effects of AEDs	0.7	2.1	13.8	41.2	42.1	1.0	5.9	28.7	51.5	13.9	1.3	3.3	25.8	50.3	19.2	56.52	<.001
Genetic etiology	0.5	6.7	16.7	30.7	45.3	0.0	11.0	19.0	30.0	40.0	2.0	8.6	26.5	40.4	22.5	31.52	<.001
Nongenetic etiology	0.4	6.4	19.1	41.5	32.6	1.0	8.0	31.0	32.0	28.0	2.0	6.0	35.8	45.7	20.5	20.44	.01
Non‐AED management	1.6	3.7	16.3	38.9	39.6	0.0	11.9	29.7	36.6	21.8	1.3	11.3	22.0	51.3	14.0	61.12	<.001
Seizure detection	0.9	7.2	23.3	37.8	30.7	1.0	10.9	14.9	45.5	27.7	2.0	13.9	40.4	31.1	12.6	44.46	<.001
Diagnosing epilepsy	0.4	7.1	23.1	35.4	34.0	0.0	14.9	24.8	40.6	19.8	1.3	6.7	16.0	48.7	27.3	24.87	.01
Lifespan	1.1	7.6	22.3	37.9	31.0	1.0	11.0	45.0	36.0	7.0	2.0	13.9	33.1	41.1	9.9	61.22	<.001
Seizure classification	0.7	8.5	25.4	44.2	21.2	0.0	7.9	20.8	45.5	25.7	1.3	6.7	31.3	43.3	17.3	6.85	.55
Prognosis	1.1	7.5	27.0	42.6	21.9	1.0	6.9	33.7	41.6	16.8	1.3	5.3	27.8	45.0	20.5	3.60	.89
Comorbidities	0.7	9.8	30.0	41.8	17.7	0.0	21.8	40.6	33.7	4.0	1.3	10.6	38.4	39.1	10.6	31.53	<.001
Anxiety	1.1	10.9	25.0	38.6	24.5	2.0	30.0	33.0	31.0	4.0	2.0	16.7	36.7	38.7	6.0	66.40	<.001
Hyperactivity	3.8	19.3	34.1	28.5	14.3	3.0	38.6	36.6	20.8	1.0	6.1	32.0	44.2	16.3	1.4	59.37	<.001
Attention	2.6	19.0	34.7	27.9	15.7	3.0	35.0	44.0	16.0	2.0	8.1	29.7	44.6	15.5	2.0	65.94	<.001
Physical capabilities	1.8	12.6	26.4	40.3	18.9	1.0	28.7	40.6	28.7	1.0	3.3	22.7	44.7	23.3	6.0	74.78	<.001
Social interactions	2.3	20.6	34.4	28.0	14.7	3.0	35.6	37.6	22.8	1.0	6.0	32.2	40.9	19.5	1.3	52.82	<.001

Abbreviation: *X* ^2^, Chi‐squared test statistic.

When post hoc statistical comparisons were conducted, the groups that differed the most in their importance rating were owners vs GP vets (Table 6), who significantly differed in their rating of 16 research areas. Owners considered 15 of these 16 areas more important than GP vets, with GP vets only considering the ways IE can be better and more quickly diagnosed as significantly more important than owners did. Owners also differed from neurologists in the importance rating of 11 research areas, with owners considering all areas more important than neurologists. Finally, GP vets and neurologists differed in their perceived importance of 4 research areas: GP vets considered the ways IE can be better and more quickly diagnosed as significantly more important than neurologists, whereas neurologists considered the development of new antiepileptic medication, identifying the genetic causes of IE, and ways to detect epileptic seizures through development of wearable technology more important than GP vets (Table 6).

**TABLE 6:** Pairwise comparisons among neurologists (n = 101), general practice (GP) vets (n = 152), and owners (n = 575) in the perceived importance future research priorities for idiopathic epilepsy (IE) in dogs. Data from 2016 and 2020 are pooled. The 2 variables where no differences were detected among any of the 3 groups (before adjustment) have been omitted

Research area	GP vets (N = 152) vs neurologists (N = 101)			Owner (N = 575) vs GP vets (N = 152)			Owner (n = 575) vs neurologists (n = 101)		
	*X* ^2^	*P*	Higher rating	*X* ^2^	*P*	Higher rating	*X* ^2^	*P*	Higher rating
Existing AEDs	2.37	.80	NA	14.24	.01	Owner	10.24	.07	NA
New AEDs	19.94	<.001	Neurologist	46.44	<.001	Owner	2.70	.75	NA
Vet education	0.18	.99	NA	56.93	<.001	Owner	42.10	<.001	Owner
Adverse effects of AEDs	2.35	.80	NA	31.99	<.001	Owner	37.03	<.001	Owner
Genetic etiology	11.89	.04	Neurologist	29.95	<.001	Owner	4.00	.55	NA
Nongenetic etiology	5.63	.34	NA	14.71	.01	Owner	9.78	.08	NA
Non‐AED management	9.16	.10	NA	42.50	<.001	Owner	31.04	<.001	Owner
Seizure detection	25.46	<.001	Neurologist	37.32	<.001	Owner	7.50	.19	NA
Diagnosing epilepsy	11.42	.04	GP vet	11.91	.04	GP vets	14.41	.01	Owner
Lifespan	4.08	.54	NA	33.32	<.001	Owner	36.76	<.001	Owner
Comorbidities	10.95	.05	NA	11.21	.05	Owner	30.83	<.001	Owner
Anxiety	6.59	.25	NA	29.60	<.001	Owner	43.25	<.001	Owner
Hyperactivity	6.97	.22	NA	36.73	<.001	Owner	33.33	<.001	Owner
Attention	3.96	.56	NA	50.19	<.001	Owner	29.23	<.001	Owner
Physical capabilities	8.34	.14	NA	44.54	<.001	Owner	42.01	<.001	Owner
Social interactions	3.94	.56	NA	24.65	<.001	Owner	24.84	<.001	Owner

Abbreviation: *X* ^2^, Chi‐squared test statistic.

#### 3.3.2 Ranking of priorities

When owners, GP vets and neurologists ranked research areas from 1 to 18, research areas ranked highest were development of new antiepileptic medication (median rank, 5.0 [2.0‐9.0]), identifying the genetic causes of IE (median rank, 7.0 [3.0‐11.75]), and nondrug management of IE such as diet (median rank, 7.0 [3.0‐12.0]) (Table 7). Six research areas significantly changed between 2016 and 2020 (Table 7). Three research areas were ranked higher in 2020 than 2016: nondrug management of IE such as diet (median rank, 7.0‐6.0), improving existing drug management of IE (median rank, 5.0‐4.0), and ways to detect epileptic seizures through development of wearable technology (median rank, 9.0‐7.0). In contrast, 3 research areas were ranked lower in 2020 than 2016: adverse effects of seizure medication and why they occur (median rank, 6.0‐7.0), nongenetic causes for epilepsy (median rank, 8.0‐9.0), and the impact of IE on dogs' physical capabilities (median rank, 12.0‐13.0).

**TABLE 7:** Owner, general practice (GP) vet, and neurologist ranking of future research priorities for idiopathic epilepsy (IE) in dogs. Research areas were ranked from 1 (top priority) to 18 (lowest priority) in 2016 and 2020 and are presented from highest to lowest ranking research areas

Research area	Overall (n = 824)	Median rank 2016 [IQR] (n = 414)	Median rank 2020 [IQR] (n = 414)	Mann‐Whitney	*P*
New AEDs	5.0 [2.0‐9.0]	5.0 [2.0‐9.0]	4.0 [2.0‐9.0]	84 206.0	.66
Genetic etiology	7.0 [3.0‐11.75]	6.0 [2.75‐11.0]	7.0 [3.0‐12.0]	92 024.0	.07
Non‐AED management	7.0 [3.0‐12.0]	7.0 [4.0‐12.0]	6.0 [3.0‐11.0]	78 073.0	.03
Existing AEDs	7.0 [4.0‐11.0]	5.0 [2.0‐9.0]	4.0 [2.0‐8.0]	77 372.5	.02
Adverse effects of AEDs	7.0 [4.0‐11.0]	6.0 [4.0‐10.0]	7.0 [4.0‐11.0]	92 569.0	.04
Vet education	7.0 [4.0‐12.0]	7.0 [4.0‐12.0]	7.0 [4.0‐12.0]	88 748.0	.37
Diagnosing epilepsy	8.0 [4.0‐12.0]	8.0 [4.0‐11.0]	8.0 [4.0‐12.0]	86 282.0	.87
Seizure detection	8.0 [4.0‐13.0]	9.0 [5.0‐14.0]	7.0 [4.0‐12.0]	73 518.0	<.001
Nongenetic etiology	8.0 [5.0‐13.0]	8.0 [5.0‐12.0]	9.0 [5.0‐13.25]	94 752.0	.01
Seizure classification	9.0 [5.0‐13.0]	9.0 [6.0‐13.0]	9.0 [5.0‐13.0]	81 613.0	.23
Prognosis	10.0 [7.0‐14.0]	10.0 [7.0‐13.0]	10.0 [7.0‐14.0]	88 946.0	.34
Lifespan	11.0 [7.0‐14.0]	11.0 [7.0‐14.0]	11.0 [7.0‐14.0]	83 594.0	.54
Comorbidities	11.0 [7.0‐14.0]	7.0 [11.0‐14.0]	10.0 [7.0‐14.0]	84 198.0	.66
Anxiety	12.0 [8.0‐15.0]	12.0 [8.0‐15.0]	12.0 [8.0‐15.0]	82 693.0	.38
Physical capabilities	12.0 [8.0‐15.0]	12.0 [8.0‐15.0]	13.0 [9.0‐16.0]	92 994.0	.03
Attention	14.0 [10.0‐16.0]	14.0 [10.0‐16.0]	13.0 [10.0‐16.0]	83 154.0	.46
Hyperactivity	14.0 [10.25‐16.0]	14.0 [10.0‐17.0]	14.0 [11.0‐16.0]	84 322.0	.69
Social interactions	14.0 [10.0‐17.0]	14.0 [10.0‐17.0]	14.0 [10.0‐17.0]	88 259.0	.46

Abbreviations: AED, antiepileptic drug; IQR, interquartile range; MW, Mann‐Whitney *U* test result.

Differences in ranking of research priorities between respondent groups were identified for 14 of the 18 research areas, with only 4 areas consistently ranked between groups: improving existing drug management of IE, nondrug management of IE such as diet, the effect of IE on other diseases (both pre‐existing and new conditions), and the impact of IE on dogs' lifespans (Table 8).

**TABLE 8:** Differences in the ranking of future research priorities for idiopathic epilepsy (IE) in dogs among neurologists (n = 101), general practice (GP) vets (n = 152), and owners (n = 575). Research areas were ranked from 1 (top priority) to 18 (lowest priority) and data from 2016 and 2020 are pooled

Research area	Owners (n = 575)	Neurologists (n = 101)	GP Vets (n = 152)	Kruskall‐Wallis	
	Median [IQR]	Median [IQR]	Median [IQR]	*F*	*P*
Existing AEDs	4.0 [2.0‐9.0]	4.0 [2.0‐7.0]	4.0 [2.0‐8.0]	5.97	.05
New AEDs	4.0 [2.0‐10.0]	4.0 [1.0‐7.0]	5.0 [3.0‐8.0]	7.89	.02
Vet education	7.0 [4.0‐12.0]	9.0 [5.0‐13.5]	9.0 [5.0‐12.0]	11.21	.01
Adverse effects of AEDs	6.0 [4.0‐11.0]	8.0 [6.0‐11.0]	6.0 [4.0‐10.0]	8.83	.01
Genetic etiology	7.0 [3.0‐12.0]	4.0 [3.0‐10.0]	7.0 [3.0‐11.0]	8.85	.01
Nongenetic etiology	9.0 [5.0‐13.0]	8.0 [4.0‐12.0]	7.0 [4.0‐11.0]	7.52	.02
Non‐AED management	6.0 [3.0‐12.0]	7.0 [4.0‐7.0]	7.0 [3.0‐11.0]	0.01	.99
Seizure detection	8.0 [4.0‐13.0]	7.0 [3.0‐11.0]	9.0 [5.0‐14.0]	9.61	.01
Diagnosing epilepsy	9.0 [5.0‐12.0]	7.0 [4.0‐10.5]	5.0 [2.0‐9.0]	38.45	<.001
Lifespan	10.0 [7.0‐14.0]	11.0 [8.0‐14.0]	11.0 [7.0‐14.0]	1.46	.48
Seizure classification	10.0 [6.0‐14.0]	7.0 [3.0‐10.0]	7.0 [4.0‐11.0]	50.78	<.001
Prognosis	11.0 [7.0‐14.0]	10.0 [7.0‐13.0]	9.0 [6.0‐12.0]	8.40	.02
Comorbidities	11.0 [7.0‐14.0]	11.0 [6.5‐14.0]	10.0 [7.0‐13.0]	2.76	.25
Anxiety	12.0 [7.0‐15.0]	13.0 [10.0‐15.0]	13.5 [10.0‐16.0]	18.02	<.001
Hyperactivity	14.0 [10.0‐16.0]	14.0 [12.0‐17.0]	15.0 [13.0‐17.0]	21.83	<.001
Physical capabilities	12.0 [8.0‐15.0]	15.0 [11.0‐17.0]	14.0 [11.0‐16.0]	47.95	<.001
Attention	13.0 [9.0‐16.0]	14.0 [11.5‐16.0]	15.0 [11.25‐16.0]	15.20	<.001
Social interactions	14.0 [9.0‐17.0]	15.0 [13.0‐17.0]	15.0 [12.0‐17.0]	15.15	<.001

Abbreviations: AED, antiepileptic drug; IQR, interquartile range; MW, Mann‐Whitney *U* test result.

When post hoc statistical comparisons were conducted, the groups with the greatest differences in ranking were owners vs neurologists, who significantly differed in 9 of the 18 research areas. Neurologists ranked 4 areas as higher priorities than owners: development of new antiepileptic medication, identifying the genetic causes IE, ways to detect epileptic seizures through development of wearable technology and how different types of epileptic seizures are classified. In contrast, owners ranked 5 areas as higher priorities than neurologists: ways to improve the education of vets regarding IE, adverse effects of seizure medication and why they occur, the impact of IE on dogs' anxiety, physical abilities, and social interactions (Table 9). Owners also differed from GP vets in their ranking of 7 priorities. General practice vets ranked 3 areas as higher priorities than owners: ways IE can be better and more quickly diagnosed, how different types of epileptic seizures are classified, and what IE means in terms of prognosis. In contrast owners, ranked 4 areas as higher priorities than GP vets, all of which fell in the category of behavioral comorbidities: the impact of IE on dogs' anxiety, hyperactivity, attention/concentration levels, and physical capabilities.

**TABLE 9:** Pairwise comparisons neurologists (n = 101), general practice (GP) vets (n = 152), and owners (n = 575) in the ranking of future research priorities for idiopathic epilepsy (IE) in dogs. Significance values have been adjusted by the Bonferroni correction for multiple tests; the 4 variables where no differences were detected among any of the 3 groups (before adjustment) have been omitted

Research area	GP vets vs neurologists			Owner vs GP vets			Owner vs neurologists		
	Mann‐Whitney	Adj. *P*	Higher ranking	Mann‐Whitney	Adj. *P*	Higher ranking	Mann‐Whitney	Adj. *P*	Higher ranking
New AEDs	−83.46	.02	Neurologist	−21.82	.93	NA	−61.64	.04	Neurologist
Vet education	18.21	1.00	NA	−52.02	.05	NA	70.33	.02	Owner
Adverse effects of AEDs	84.33	.02	GP Vet	13.54	1.00	NA	70.79	.02	Owner
Genetic etiology	−70.33	.07	NA	5.95	1.00	NA	−76.28	.01	Neurologist
Nongenetic etiology	6.23	1.00	NA	51.74	.05	NA	−45.50	.23	NA
Seizure detection	−94.34	.01	Neurologist	−31.13	.46	NA	−63.21	.04	Neurologist
Diagnosing epilepsy	90.54	.01	GP Vet	132.34	<.001	GP Vet	−43.78	.27	NA
Seizure classification	−23.13	1.00	NA	118.39	<.001	GP Vet	−141.52	<.001	Neurologist
Prognosis	40.83	.55	NA	62.64	.01	GP Vet	−21.81	1.00	NA
Anxiety	11.41	.71	NA	−80.67	<.001	Owner	69.26	.02	Owner
Hyperactivity	−44.72	.43	NA	−97.48	<.001	Owner	52.86	.12	NA
Attention	−17.61	1.00	NA	−76.35	.001	Owner	58.73	.07	NA
Physical capabilities	41.65	.52	NA	−105.57	<.001	Owner	147.22	<.001	Owner
Social interactions	36.65	.69	NA	−51.87	.05	NA	88.52	.01	Owner

Finally, neurologists differed from GP vets in the ranking of 4 research areas. Neurologists ranked 2 areas as higher priorities than GP vets: development of new antiepileptic medication and ways to detect epileptic seizures through development of wearable technology. In contrast, GP vets ranked 2 areas as higher priorities than neurologists: the adverse effects of seizure medication and why they occur, and ways IE can be better and more quickly diagnosed (Table 9).

#### 3.3.3 Perception of nondrug management of IE

Of 10 nondrug therapies, the 5 rated to have the highest potential positive impact on IE management were: behavioral management, gene editing, CBD oil supplementation, MCT oil supplementation, and epilepsy surgery (Table 10). Differences in the ratings of nondrug therapies were detected for 7 of the 10 nondrug therapies, with only epilepsy surgery, TMS, and gene editing rated consistently between GP vets, owners, and neurologists (Table 11). Owners and neurologists differed in their rating of the potential impact of 7 nondrug therapies, with owners considering all therapies to have a higher potential positive impact on epilepsy management (Table 12). Similarly, owners rated 3 nondrug therapies to have a higher potential positive impact on epilepsy management than GP vets, namely raw food diet, VNS, and DBS. Differences between GP vet and neurologists in the evaluation of the impact of nondrug management was limited to hypoallergenic diets, which GP vets rated more highly.

**TABLE 10:** Perceived potential positive impacts of emerging nondrug therapies upon the management of idiopathic epilepsy (IE) in dogs. Rated by n = 273 owners, n = 68 general practice (GP) vets, and n = 73 neurologists in 2020

Nondrug treatment	Total (N)	Perceived positive impact on epilepsy management (%)				
		No impact (0)	Little impact (1)	Some impact (2)	Great impact (3)	Major impact (4)
Behavioral management	401	3.0	14.5	39.4	26.9	16.2
Gene editing	284	7.7	17.3	35.2	22.2	17.6
Cannabidiol oil	385	5.7	17.9	39.5	22.1	14.8
Medium‐chain triglyceride oil	312	4.5	12.2	49.7	21.5	12.2
Epilepsy surgery	323	7.4	24.8	35.6	21.4	10.8
Vagus nerve stimulation	244	4.9	21.7	51.2	15.2	7.0
Deep brain stimulation	219	5.9	25.6	45.7	14.6	8.2
Hypoallergenic diet	379	14.0	24.8	43.3	11.6	6.3
Raw food diet	393	31.8	23.7	27.2	10.7	6.6
Transcranial magnetic stimulation	199	7.5	28.6	46.7	11.6	5.5

**TABLE 11:** Differences among owners (n = 273), general practice (GP) vets (n = 68), and neurologists (n = 73) perceptions of the potential positive impacts of emerging nondrug therapies upon the management of idiopathic epilepsy (IE) in dogs

Nondrug treatment	Owners (%)					Neurologists (%)					GP Vets (%)					Statistical test	
	0	1	2	3	4	0	1	2	3	4	0	1	2	3	4	*X* ^2^	*P*
Cannabidiol oil	5.6	13.5	37.1	24.3	19.5	4.1	34.2	38.4	19.2	4.1	8.2	16.4	50.8	16.4	8.2	29.94	<.001
Medium‐chain Triglyceride oil	3.9	7.8	52.0	22.9	13.4	5.5	24.7	49.3	13.7	6.8	5.0	10.0	43.3	26.7	15.0	18.66	.02
Raw food diet	16.1	24.4	35.0	14.6	9.8	69.9	19.2	8.2	2.7	0.0	50.0	25.8	18.2	4.5	1.5	99.52	<.001
Hypoallergenic diet	9.9	19.3	48.1	14.0	8.6	31.0	33.8	29.6	5.6	0.0	10.8	35.4	40.0	9.2	4.6	41.37	<.001
Vagus nerve stimulation	4.8	11.3	52.4	20.2	11.3	5.6	36.1	47.2	8.3	2.8	4.2	27.1	54.2	12.5	2.1	25.42	<.001
Deep brain stimulation	1.8	17.1	52.3	18.0	10.8	8.7	37.3	34.8	13.0	5.8	12.8	28.2	46.2	7.7	5.1	21.34	.01
Gene editing	7.0	13.3	35.4	23.4	20.9	7.5	23.9	37.3	16.4	14.9	10.2	20.3	32.2	25.4	11.9	8.02	.43
Behavioral management	2.7	12.6	35.9	29.4	19.5	4.2	25.0	45.8	16.7	8.3	3.0	10.4	46.3	28.4	11.9	18.29	.02
Transcranial magnetic stimulation	5.6	21.1	50.0	13.3	10.0	7.1	37.1	44.3	11.4	0.0	12.8	30.8	43.6	7.7	5.1	13.80	.09
Epilepsy surgery	9.0	19.0	38.1	20.1	13.8	5.6	34.7	29.2	25.0	5.6	4.8	30.6	35.5	21.0	8.1	13.30	.10

**TABLE 12:** Pairwise comparison of perceived positive impact of nondrug therapies on dog idiopathic epilepsy (IE) management compared among general practice (GP) vets (n = 68), neurologists (n = 73), and owners (n = 273). Results are only presented for the 7 of the 10 treatment areas where overall significant differences in impact rating were detected between groups. The respondent group who rated the impact on epilepsy management more highly is highlighted in each pair where significant differences (*P* < 0.05) are detected

Nondrug treatment	GP vets vs neurologists			Owners vs GP vets			Owners vs neurologists		
	*X* ^2^	*P*	Higher impact	*X* ^2^	*P*	Higher impact	*X* ^2^	*P*	Higher impact
Cannabidiol oil	7.23	.12	NA	8.16	.09	NA	22.58	<.001	Owner
Medium‐chain triglyceride oil	9.10	.06	NA	1.40	.84	NA	16.05	.01	Owner
Raw food diet	7.01	.14	NA	39.41	<.001	Owner	86.71	<.001	Owner
Hypoallergenic diet	11.47	.02	GP Vet	8.54	.07	NA	35.41	<.001	Owner
Vagus nerve stimulation	1.67	.80	NA	10.04	.04	Owner	22.11	<.001	Owner
Deep brain stimulation	2.56	.63	NA	12.50	.01	Owner	16.46	.01	Owner
Behavioral management	6.80	.15	NA	3.44	.49	NA	15.12	.01	Owner

## 4 DISCUSSION

Research into IE in dogs is a growing and multifaceted area of veterinary medicine. This study has considered future research priorities in IE in dogs, drawing opinions from 3 major stakeholder groups involved in this disorder: people who own or have previously owned a dog with IE, general practice veterinary surgeons, and neurology specialist in referral practice. This multistakeholder prioritization activity is novel in veterinary medicine but this type of activity is an established practice in human medicine. An example of this is The James Lind Alliance that brings patients, carers, and clinicians together as priority sharing partnerships, aiming to ensure that research is targeted at questions that matter to these interested parties, and that agreement on those areas that deserve priority attention are highlighted.^40^ Although the same formal methodology was not conducted for this study, it demonstrates the feasibility of this type of study in veterinary medicine and a foundation for future work.

In our prioritization exercise, when both relatively ranked and rated for absolute importance, the areas that garnered the most favor for future research focused on ASD management of IE, both improving existing drug management of IE and the development of new antiepileptic medication. Given that ASDs are the mainstay of IE management,^24^ this is perhaps unsurprising, and the need for more research in this area likely reflects some of the inherent issues faced by clinicians and owners alike regarding both true drug‐resistance^41^ and continued seizure activity despite ASD treatment, that might require trial‐and‐error amendments to treatment schedules (eg, the use of polytherapy^42^). Similar findings are seen in human epilepsy, where ASD development also remains a key research benchmark,^43^ with a strong push to develop new ASD therapies.^44^ In Europe, the number of available ASDs for dog IE as either first line or adjunctive therapies has grown in recent years, with new therapies including imepitoin.^45^ However, as discussed in the recent IVETF consensus statement on the medical treatment of IE, the use of ASDs is complex, with several variables modifiable in their use, including when to start treatment, which drug is best used initially, which adjunctive ASD can be advised if treatment with the initial drug is unsatisfactory, and when treatment changes should be considered.^24^ The development and approval of new ASDs is likely to be a long‐term activity, given that all but 1 of the ASDs licensed for dogs are derived from human medicine, and many novel human ASDs are unsuitable for dogs.^45^ Consequently, studying the clinical effects of existing ASDs in high quality studies could contribute to the refinement of their use. As highlighted in a recent systematic review of ASD efficacy, most evidence on ASDs for dog IE are derived from nonblinded nonrandomized uncontrolled trials and case series, with many using subjective outcome measures.^46^ Conducting high quality trials to determine the most efficacious treatments, or treatment combinations, and moving toward a personalized medicine approach has the potential to improve ASD efficacy in dogs as well as humans. In human medicine, proposed strategies for this include creating personalized disease models for drug screening to identify targeted and effective treatment, using stem cell technologies and machine learning.^47^

A further area deemed an important research priority was ways to improve the education of vets regarding IE, an area that owners rated as more important than either vets and neurologists. Veterinarian‐reported deficits in epilepsy knowledge were identified in a recent study of Dutch first‐opinion practitioners, particularly regarding differentiation of epilepsy from other paroxysmal disorders, between epilepsy types and between epileptic seizure types.^48^ In addition, only moderate levels of confidence were reported for knowing when to adjust ASD treatment.^48^ In a recent qualitative study of owners experiences managing a dog with IE, some interviewees expressed feelings of stress and uncertainty regarding their dog's disease process, which sometimes led them to use the internet to perform self‐directed research on their dog's condition.^21^ This supports previous research indicating that companion animal owners who were uncertain regarding recommendations from their veterinary surgeon were more likely to perform self‐directed research.^49^ Although in the Dutch study, vets felt confident in communicating with owners in terms of offering comfort, explaining epilepsy as a diagnosis and its prognosis to owners,^48^ the results presented here, where owners considered veterinary education a high priority, combined with recent findings regarding distress and uncertainty in this ownership group,^49^ might indicate disparities in the perceived effectiveness of veterinary communication between vets and owners. As such, finding ways to ensure that undergraduate and postgraduate education regarding epilepsy is sufficient for vets to feel confident in their epilepsy diagnostic skills, but also their ability to communicate effectively with owners of dogs with epilepsy, is likely to benefit affected dogs, owners, and vets alike.

Identifying the genetic causes of IE was consistently considered an important area of future research, which was particularly valued by neurologists. The genetic understanding of IE in dogs is growing,^50^ and genetic testing as an aid to diagnosis, prognosis, and breeding decisions is available for some forms of epilepsy in several breeds^51^; however, these successes have been largely achieved in progressive myoclonic epilepsies, where reactive seizures are caused by metabolic abnormalities.^52^, ^53^, ^54^ In contrast, many studies of dog breeds with idiopathic epilepsies have failed to identify genes or loci of interest.^51^ This slow progress suggests that IE in dogs, as seen in human epilepsies, is likely an extremely complex genetic picture, which is almost certainly polygenic with potential gene‐environmental interactions. Although both challenging and expensive studies, successes in gene identification could give hope to dog breeders aiming to eradicate epilepsy in their breed, as has been attempted with some progressive myoclonic epilepsies,^52^ and are thus valiant pursuits within veterinary medicine.

An emerging area of importance ranked as the third highest research priority and increasing in rank between 2016 and 2020 was nondrug management of IE. This area was considered by owners to have a higher potential positive impact than perceived by GP vets or neurologists, which might reflect their frustration with current ASD‐based management strategies, with previous research finding owners are motivated to use a range of dietary supplements (with or without evidence of efficacy) to reduce their dog's seizure frequency and severity, even when treated with ASDs.^55^ Nonpharmacological treatment options for IE are becoming increasingly important in human medicine as well as veterinary medicine, with a range of novel options for humans now being trialed in dogs.^34^ Those considered to have the most positive impact on IE management included noninvasive methods (eg, behavioral management, use of dietary supplements such as CBD and MCT oils) and invasive methods (eg, epilepsy surgery), with varying levels of existing evidence for the use of these therapies.

Evidence for the efficacy of MCT supplementation to the diets of dogs with IE is increasing, with reductions in epileptic seizures, behavioral and cognitive comorbidities both when combined within a kibble diet^56^, ^57^, ^58^ and when added as a supplement to a dog's base diet.^59^, ^60^ Understanding the mechanisms behind these positive effects (eg, impacts on the dog microbiome and metabolome^61^), and identifying profiles of dogs most likely to respond to this dietary intervention is a future priority for MCT research. Evidence for CBD oil supplementation in dogs with IE is in its relative infancy, with a preliminary randomized control study indicating that although the CBD‐treated group had a 33% decrease in the group median for mean monthly seizure frequency compared with the placebo‐treated group, the proportion of dogs considered treatment “responders” (ie, ≥50% decrease in seizure activity) was similar between the 2 groups.^62^ Given the popularity of its use with owners even without strong efficacy data,^55^ this is an area where further research is urgently needed, ideally from larger‐scale studies over a longer time frame.^63^ The use of behavioral management of epilepsy is a commonplace in human epilepsy, including trigger management, stress‐reduction therapies, and specific relaxation‐based therapies, and have been suggested as a novel addition to the epilepsy management tool kit in dogs.^64^ With seizure triggers^5^, ^65^ and chronic stress^66^ increasingly recognized in dogs, devising evidence‐based behavioral interventions and conducting high‐quality clinical trials to test their efficacy is of priority. Surgery is the treatment of choice in human epilepsy, particularly in cases with a well‐defined focal onset where at least 2 ASDs have failed to provide control. A range of surgery types exist that can be curative or palliative,^67^ including resective techniques to attempt to remove the epileptogenic focus without damaging healthy cortical tissue and eliminate epileptic seizures entirely. To date, attempts to perform epilepsy surgery in dogs have been hampered by challenges in localizing the origin of epileptic seizures, and thus further research to improve the localization of the epileptogenic focus are needed.^68^ Finally, gene editing was rated highly as a nondrug treatment for IE. This area of research has not yet been explored for IE in dogs, and is in its infancy in human medicine, but has been identified as an emerging therapeutic approach for drug‐resistant epilepsy management, particularly the use of clustered regularly interspaced short palindromic repeats (CRISPR) technology.^69^ This is likely to be a longer‐term research goal, given there are still some challenges with CRISPR regarding efficiency and accuracy, and further studies are needed to verify its safety before clinical applications in people or dogs.

Our findings indicate agreement between stakeholders for some areas, but disparities for others, particularly between owners and the 2 veterinary stakeholder groups. This is not entirely unexpected, with studies comparing concerns regarding epilepsy between human epilepsy patients and their doctors identifying disparities in priorities between these groups, particularly for cognitive comorbidities such as memory problems.^70^ Indeed, in the current study, comorbidities were ranked more highly by owners than GP vets and neurologists. In the aforementioned human study, doctors were found to focus more on clinical issues, where patients focus was more on “life issues”.^70^ Although many “lifestyle issues” associated with epilepsy in people do not translate to dogs (eg, restrictions on driving), owners also appeared more focused on day‐to‐day issues in the current study, ranking areas such as the physical, social, and behavioral capabilities of their dog, and adverse effects of ASDs more highly than GP vets or neurologists. Similarly, in an experiment that compared the ranking of people with epilepsy and neurologists on the attributes of ASDs,^71^ patients and neurologists ranked seizure control as the most important attribute, but patients prioritized ASD adverse effects that might impact their QoL, including ataxia, lethargy, and psychiatric effects more highly than their neurologist.^71^ In the current study, GP vets placed more emphasis on clinical issues such as IE diagnosis and prognosis, and neurologists placed more focus on the use of cutting‐edge technologies such as understanding the genetic underpinnings of IE and using technology to detect epileptic seizures. The latter might reflect a longer‐term approach to IE research, adopting approaches from human medicine that specialists might have greater exposure to, given additional time to focus on epilepsy and other neurological disorders compared to GP vets, including engagement with research as part of their specialist credentials.

Although providing novel insights, this study has a number of limitations that should be acknowledged, including a self‐selecting, and relatively small sample for some of the stakeholder groups, which might be biased toward respondents with a particular interest in IE in dogs, which might not be representative of their wider group. Given there are around 20 000 vets in the United Kingdom, 53000 UK dogs diagnosed with IE (0.6%^72^ of the likely 8.9 million dogs in the general population) and 217 ECVN recognized specialists,^73^ the generalizability of these results could be limited, but offer a starting point in understanding differences between these groups. Specific biases include a large proportion of owners in both the 2016 and 2020 sample had interacted with a specialist neurologist as well as a GP vet during their dog's management. This might have affected their perceptions of priority areas given they could have been exposed to wider and deeper knowledge of IE in dogs because of the potentially greater time and knowledge a referral consultation can offer. In addition, the research areas offered for prioritization were generated by the author team and thus might reflect some of their own inherent biases, and indeed respondents might have been influenced by the knowledge of the research interests of the team conducting the study, or might have indeed been collaborators. In the future, a more structured Delphi approach with a range of participants could be used to promote a diversity of topics for inclusion, based on expert consensus.^74^

## CONFLICT OF INTEREST DECLARATION

Authors declare no conflict of interest.

## OFF‐LABEL ANTIMICROBIAL DECLARATION

Authors declare no off‐label use of antimicrobials.

## INSTITUTIONAL ANIMAL CARE AND USE COMMITTEE (IACUC) OR OTHER APPROVAL DECLARATION

Authors declare no IACUC or other approval was needed.

## HUMAN ETHICS APPROVAL DECLARATION

The study protocol and design were approved by the Royal Veterinary College Ethics and Welfare Committee (URN 2016 T85). The data collected in this trial are collated and stored at the Royal Veterinary College in London (RVC). Data was anonymized as appropriate, and only used for analysis. This manuscript was internally approved for submission (1442552).
